# Protein Translocation into the Intermembrane Space and Matrix of Mitochondria: Mechanisms and Driving Forces

**DOI:** 10.3389/fmolb.2017.00083

**Published:** 2017-12-07

**Authors:** Sandra Backes, Johannes M. Herrmann

**Affiliations:** Cell Biology, University of Kaiserslautern, Kaiserslautern, Germany

**Keywords:** brownian ratchet, disulfide bond, foldase, holdase, Mia40, mitochondria, protein import

## Abstract

Mitochondria contain two aqueous subcompartments, the matrix and the intermembrane space (IMS). The matrix is enclosed by both the inner and outer mitochondrial membranes, whilst the IMS is sandwiched between the two. Proteins of the matrix are synthesized in the cytosol as preproteins, which contain amino-terminal matrix targeting sequences that mediate their translocation through translocases embedded in the outer and inner membrane. For these proteins, the translocation reaction is driven by the import motor which is part of the inner membrane translocase. The import motor employs matrix Hsp70 molecules and ATP hydrolysis to ratchet proteins into the mitochondrial matrix. Most IMS proteins lack presequences and instead utilize the IMS receptor Mia40, which facilitates their translocation across the outer membrane in a reaction that is coupled to the formation of disulfide bonds within the protein. This process requires neither ATP nor the mitochondrial membrane potential. Mia40 fulfills two roles: First, it acts as a holdase, which is crucial in the import of IMS proteins and second, it functions as a foldase, introducing disulfide bonds into newly imported proteins, which induces and stabilizes their natively folded state. For several Mia40 substrates, oxidative folding is an essential prerequisite for their assembly into oligomeric complexes. Interestingly, recent studies have shown that the two functions of Mia40 can be experimentally separated from each other by the use of specific mutants, hence providing a powerful new way to dissect the different physiological roles of Mia40. In this review we summarize the current knowledge relating to the mitochondrial matrix-targeting and the IMS-targeting/Mia40 pathway. Moreover, we discuss the mechanistic properties by which the mitochondrial import motor on the one hand and Mia40 on the other, drive the translocation of their substrates into the organelle. We propose that the lateral diffusion of Mia40 in the inner membrane and the oxidation-mediated folding of incoming polypeptides supports IMS import.

## Introduction

**Figure 1:**
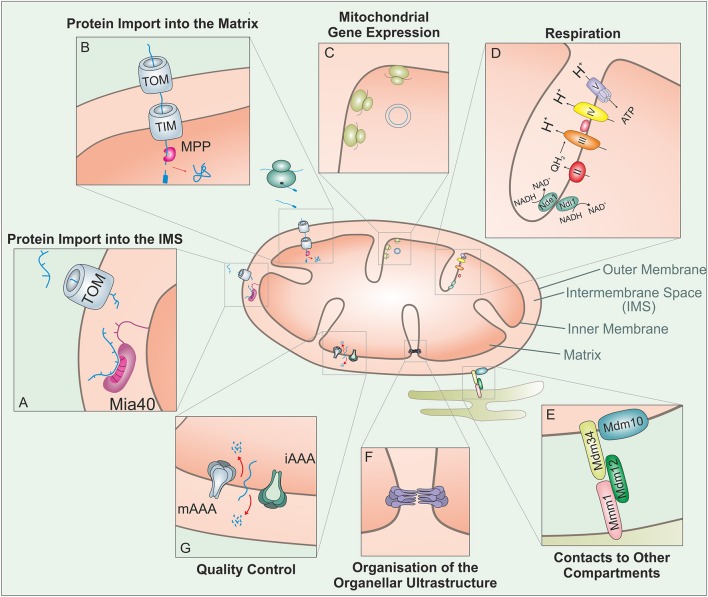
Mitochondria carry out a large variety of different biological activities. This figure shows some of these functions which are relevant in the context of this review: The vast amount of mitochondrial proteins need to be imported from the cytosol. **(A)** Proteins targeted to the IMS enter through the TOM complex and bind to the IMS receptor Mia40, which is responsible for the introduction of disulfide bonds and concomitant protein folding. **(B)** Proteins with an N-terminal MTS are guided to the matrix through the TOM and TIM complex. The mitochondrial processing peptidase (MPP) removes the MTSs from preproteins before they can fold into their native structures. **(C)** Mitochondria contain a complete genetic system for replication, transcription and translation that is entirely distinct from that in the nucleus/cytosol. The mitochondrial translation system is membrane-bound and specialized on the synthesis of a small number of very hydrophobic polypeptides. **(D)** Mitochondria contain a large number of metabolic enzymes that carry out a variety of catabolic and anabolic pathways. Of particular abundance and relevance are the complexes of the respiratory chain, which use the transfer of electrons to generate an electrochemical gradient which drives the synthesis of the vast majority of the cellular ATP. **(E)** Mitochondria interact with many other cellular compartments. The ER-mitochondria encounter structure (ERMES) tethers mitochondria to the ER, presumably to facilitate the exchange of calcium and phospholipids between their membranes. **(F)** The ultrastructure of mitochondria depends on the function of a number of protein complexes. Of particular importance is the “cristae organizing system” (MICOS) which participates in the formation of cristae junctions and contact sites of the inner and outer membrane. **(G)** Mitochondrial peptidases can regulate different mitochondrial functions through proteolytic processing and protein degradation. In addition to a number of soluble proteases, the inner membrane contains two very important ATP-hydrolysing protease complexes that belong to the AAA (ATPases associated with different cellular activities) protein family: these i-AAA and m-AAA proteases expose their proteolytic domains to the IMS and membrane sides of the inner membrane, respectively.

Mitochondria are essential organelles of eukaryotic cells that carry out a large range of different activities (Figure 1). Only a very small proportion of the ~1,000 mitochondrial proteins are synthesized by mitochondrial ribosomes (8 in baker's yeast, 13 in humans, and 67 in *Reclinomonas americana*, the organism with the most complex mitochondrial genome; Lang et al., 1997). All other proteins are synthesized in the cytosol from where they are imported into mitochondria by a *t*ranslocase in the *o*uter membrane of *m*itochondria (TOM complex) and *t*ranslocases in the *i*nner membrane of *m*itochondria (TIM complexes). Proteins of the matrix and the inner membrane, each making up about 40% of all mitochondrial proteins (Calvo et al., 2016; Morgenstern et al., 2017), employ both TOM and TIM translocases and predominantly use similar mechanisms for their import process. These proteins include many of the enzymes that catalyze the major biochemical functions of the organelle in metabolic conversions and respiration, in the biosynthesis of lipids, iron-sulfur clusters, heme, and amino acids, or in the expression of mitochondrial genes (Figure 1). Proteins of the outer membrane and the IMS, each making up about 10% of the mitochondrial proteome, use a diversity of strategies to make their way into mitochondria. Outer membrane and IMS proteins play crucial roles in the communication with the cytosol and with other mitochondrial compartments, in the uptake of metabolites, lipids, or metal ions as well as with the regulation and execution of apoptosis (Wang and Youle, 2009; Herrmann and Riemer, 2010; Aaltonen et al., 2016; Miyata et al., 2016).

In this review we focus on the import of proteins into the IMS and discuss recent studies on the mechanisms of the Mia40-mediated import reaction and compare this process to the well-studied preprotein pathway which drives the translocation of matrix and inner membrane proteins.

## Protein import into the mitochondrial matrix

Proteins destined to the mitochondrial matrix are synthesized with amino terminal presequences that serve as matrix-targeting sequences (MTSs). These sequences form amphipathic helices that vary largely in primary sequence but are characterized by a length of about 15–60 residues, a net charge of +3 to +6, the absence of negatively charged residues and a high content of hydroxylated amino acids (Vögtle et al., 2009; Calvo et al., 2017). MTSs are recognized by the receptors Tom20/Tom22 and Tom70 on the mitochondrial surface, which have considerable overlap in specificity and functionality (Brix et al., 1999; Fan et al., 2011). The binding of MTSs to the TOM receptors, particularly to Tom70, is facilitated by cytosolic chaperones of the Hsp70 and Hsp90 classes as well as by co-chaperones such as Sti1 (Young et al., 2003; Hansen et al., 2016; Hoseini et al., 2016).

**Figure 2:**
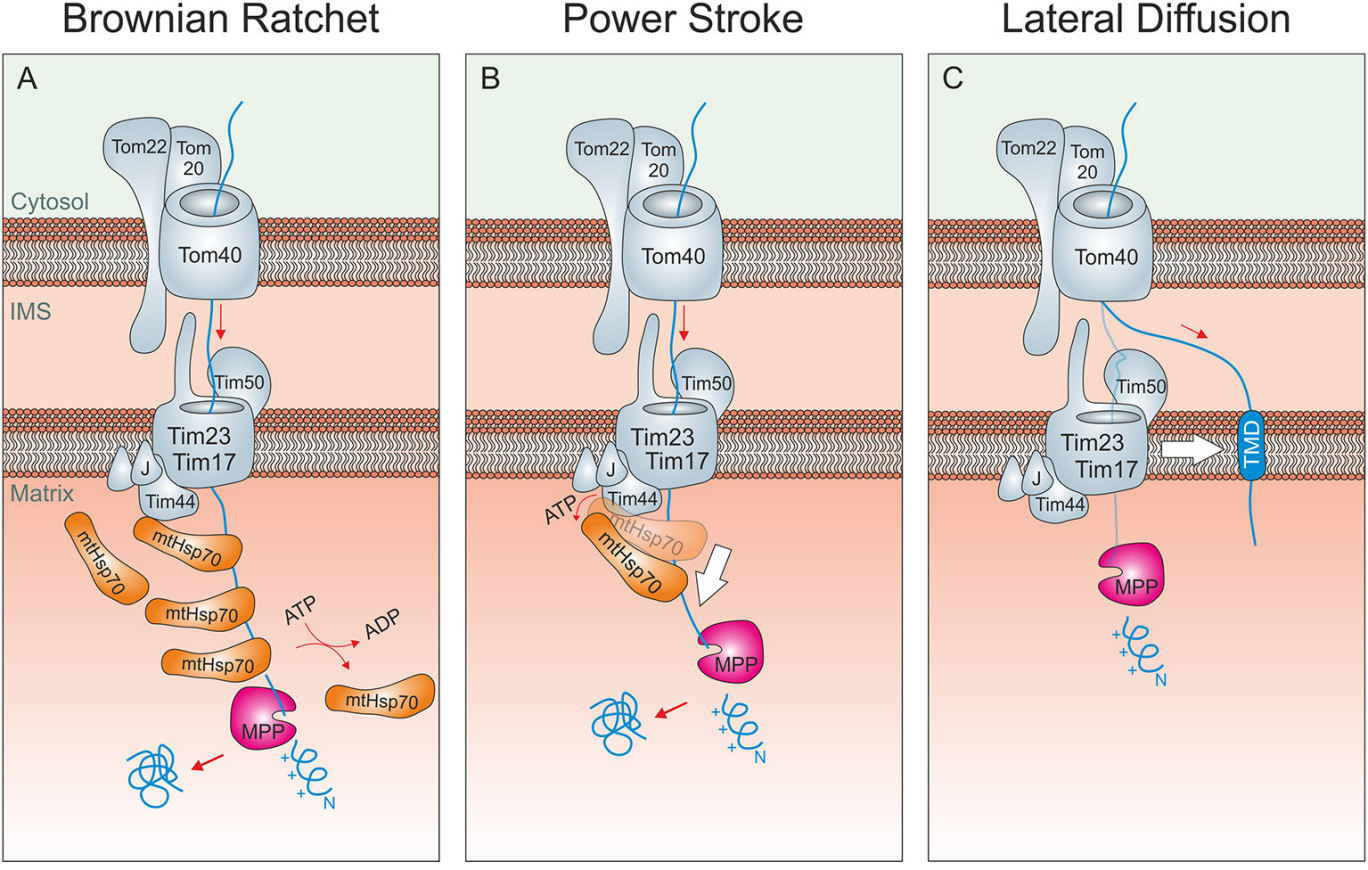
Driving forces of mitochondrial protein import. **(A)** Brownian ratchet: According to this model, the ATP-hydrolysis does not promote a mechanical pulling of the incoming polypeptide. Rather, Hsp70 molecules associated to Tim44 bind to unfolded segments of the translocating protein, preventing their backsliding and thus rectifying their Brownian spontaneous motion into a vectorial movement into the matrix. **(B)** Power stroke: It was proposed that after binding to Tim44 and the presequence of an incoming preprotein, ATP-hydrolysis in Hsp70 triggers a large conformational change within the chaperone that leads to a mechanical pulling of the preprotein into the matrix. Repeated cycles would over time drive protein-translocation in a step-wise fashion. A pulling of Hsp70 was suggested to be particularly important if a folded domain on the mitochondrial surface needs/ed to be unfolded. It should be noted that both models are not mutually exclusive. **(C)** Lateral Diffusion: A number of inner membrane proteins contain stop-anchor sequences just C-terminal to their presequences. How their C-terminal domains are transported across the outer membrane is not known. It was suggested that lateral diffusion thus the separation of TOM and TIM23 complexes might be critical here.

Following receptor binding the preproteins are threaded through the protein-conducting channel of the TOM complex that is formed by the beta barrel protein Tom40 (Shiota et al., 2015) and are subsequently transferred to the TIM23 complex of the inner membrane. This translocase comprises three essential inner membrane proteins: Tim50, Tim23, and Tim17 (Figure 2). Tim50 exposes a large domain into the IMS that promotes the transfer of preproteins from the TOM to the TIM23 translocase and regulates the gating of the TIM channel (Mokranjac et al., 2009; Schulz et al., 2011; Bajaj et al., 2014). Tim23 and Tim17 are two structurally related multi-spanning inner membrane proteins that form the protein-conducting channel of the inner membrane (Truscott et al., 2001; Meier et al., 2005; Zarsky and Dolezal, 2016). Tim17, which shows the highest degree of sequence conservation of all TOM and TIM subunits, coordinates the membrane potential-dependent opening of the channel with the binding of the import motor on the matrix site via the membrane-associated subunit Tim44 (Meier et al., 2005; Martinez-Caballero et al., 2007; Ramesh et al., 2016; Demishtein-Zohary et al., 2017; Matta et al., 2017; Ting et al., 2017). Tim44 serves as binding site for the mitochondrial Hsp70 (mtHsp70) of the matrix which binds tightly to incoming polypeptides upon hydrolysis of its bound ATP to ADP in a reaction regulated by Pam18/Tim14 and Pam16/Tim16, a J protein and a J-like protein, respectively, that are associated with the TIM23 complex. Several binding reactions of mtHsp70 molecules are normally required to drive the complete translocation of a protein into the matrix. Mitochondrial presequences are removed in the matrix by the matrix processing peptidase. Preproteins may additionally be further processed by other enzymes before the mature proteins fold into their native structures. It should be noted that there appears to be no back-translocation across the inner membrane, so that matrix proteins stay in the matrix until they are degraded. This is different for IMS proteins, for which a back-translocation into the cytosol was reported under certain conditions (Bragoszewski et al., 2015).

Many presequence-containing inner membrane proteins are integrated into the inner membrane after translocational arrest by a transmembrane domain in their structure that serves as a stop-transfer sequence. Alternatively, inner membrane proteins can be initially completely imported into the matrix from where they insert into the inner membrane in an export-like reaction (Herrmann et al., 1997; Bohnert et al., 2010). Membrane insertion from the matrix is catalyzed by the Oxa1 protein of the inner membrane that is related to the bacterial insertase YidC (Hell et al., 2001). Some inner membrane proteins, such as members of the carrier family, lack presequences but insert into the inner membrane from the IMS using an alternative TIM translocase, the TIM22 complex (Sirrenberg et al., 1996; Hasson et al., 2010).

## Energetics of the protein import into the mitochondrial matrix

The mechanistic details of the translocation process across the TOM and the TIM23 were analyzed in many studies. However, our understanding of the molecular clockwork that drives protein translocation *in vivo* is still far from complete. This owes to the facts that (i) high-resolution structures of the import machinery are not available, (ii) the components of the import machinery interact in a highly dynamic fashion, and (iii) their interaction can only be analyzed *in vitro* or in organello as lysis even with mild detergents often leads to the loss of components and prevents the analysis of their functionality.

The initial translocation reactions from receptors on the mitochondrial surface via the TOM pore and IMS-exposed domains of the import machinery (Tom22, Tim50, Tim23) to the translocation pore of the TIM23 complex is presumably driven by the affinity of presequences to hydrophobic and negatively charged surfaces on components of the import machinery (Bolliger et al., 1995; Kanamori et al., 1997; Mokranjac et al., 2009; Shiota et al., 2015; Bausewein et al., 2017). Subsequently, the membrane potential across the inner membrane can further promote the translocation of the positively charged MTSs to the negatively charged, matrix side of the inner membrane (van der Laan et al., 2007). The inner membrane pore is gated by the presequences in a complicated process that involves an intricate interplay of Tim50, Tim23, and Tim17 (Meier et al., 2005; Martinez-Caballero et al., 2007; Schulz et al., 2011; Ramesh et al., 2016; Denkert et al., 2017; Schendzielorz et al., 2017). As soon as the presequence is exposed to the matrix, it can be bound by mitochondrial Hsp70 (mtHsp70) which completes matrix translocation of preproteins.

Two not mutually exclusive models were proposed, how mtHsp70 and other subunits of the mitochondrial import motor promote unidirectional translocation into the matrix (Figures 2A,B). The pros and cons of both models have been extensively debated in many excellent studies and reviews in the past and will therefore not be repeated in detail here (Glick, 1995; Matlack et al., 1999; Voisine et al., 1999; Neupert and Brunner, 2002; Okamoto et al., 2002; Slutsky-Leiderman et al., 2007; Yamano et al., 2008). According to the Brownian ratchet model, repetitive binding of several mtHsp70 proteins prevents backsliding of intermediates. The energy for this translocation reaction initially comes from the Brownian movement of the incoming chain that is rectified by mtHsp70. According to the alternative power stroke model, mtHsp70 actively pulls on the incoming polypeptides to drive their import in a step-wise fashion. Since the intramolecular movements of mtHsp70 are rather small, it is unlikely that they could processively run precursors into the matrix. However, small movements might help to unfold cytosolic domains of stalled import intermediates while the main driver of translocation is still rectified diffusion. From this perspective, the power stroke model can be seen as an extension rather than an alternative to the Brownian Ratchet model. The tight contact of mtHsp70 to the protein-conducting channel of the TIM23 translocase, which is critical for both mechanisms, is mediated by Tim44, Pam18/Tim14, and Pam16/Tim16 (Demishtein-Zohary et al., 2017; Matta et al., 2017; Ting et al., 2017).

An interesting variation of the Brownian Ratchet model was recently proposed (De Los Rios et al., 2006; Finka et al., 2015). Though this model is called entropic pulling, no power stroke is involved here. Rather, changes in the conformational freedom of the components of the import motor and the incoming polypeptide forcefully drive the unfolding and translocation of precursor proteins in an ATP-dependent reaction. Whether the hypothesis of entropic pulling really settles the debate between translocation by Brownian Ratchet and by power stroke is still open. However, it certainly is very attractive since it naturally integrates the ratcheting property of the Brownian Ratchet model and the active pulling action of the power stroke model in a single framework. The import motor is unable to drive the translocation of inner membrane proteins with amino terminal transmembrane domains. It is not clear how C-terminal domains of these proteins are driven across the TOM complex but it was proposed that the lateral diffusion of the transmembrane domains drives this process (Figure 2C). Nonetheless, it is difficult to envision how diffusion alone shall be sufficient to unfold domains in the cytosol in order to allow their passage through the TOM pore. This is one of the open questions in the field that still awaits to be answered.

## The mitochondrial disulfide relay

The IMS is a small compartment that is enclosed by the outer and inner membrane of mitochondria. Proteomic studies identified about 50 and 130 different IMS proteins in yeast and mammalian mitochondria, respectively (Vögtle et al., 2012; Hung et al., 2014). Some of these proteins, particularly those of larger mass and multi-domain organization are synthesized with “bipartite presequences,” i.e., amino terminal targeting signals consisting of an MTS followed by a stop-transfer sequence that is cleaved off after translocation thus giving rise to a mature soluble IMS protein. Examples for IMS proteins with bipartite presequences include proapoptotic factors such as Smac/Diablo (Burri et al., 2005), apoptosis-inducing factor (AIF) (Hangen et al., 2015), or endonuclease G (Ohsato et al., 2002) as well as enzymes such as cytochrome *b*_2_ (Glick et al., 1992), Mgm1 (Herlan et al., 2004), or cytochrome *c* peroxidase (Michaelis et al., 2005).

Most proteins of the IMS lack presequences and their amino termini do not share any characteristics with those of matrix proteins. Many of these proteins are of relatively low mass (7–25 kDa) and of rather simple structure (most frequent is a simple helix-loop-helix organization). Many of these proteins appear to make use of high-affinity binding sites in the IMS that are crucial for their translocation through the protein-conducting channel of the TOM complex. The most abundant IMS protein, cytochrome *c*, employs its hemylating enzyme cytochrome *c* heme lyase as a trans-site receptor (Nargang et al., 1988; Nicholson and Neupert, 1989; Dumont et al., 1991). Apocytochrome *c* can cross the outer membrane through the TOM complex in both directions. In the IMS, cytochrome *c* heme lyase incorporates a heme group into apocytochrome *c* and thereby triggers its stable folding rendering holocytochrome *c* unable to retro-translocate through the TOM complex. In this reaction, cytochrome *c* heme lyase functions both as a receptor and as a converting enzyme that catalyzes the stable folding of cytochrome *c*.

**Figure 3:**
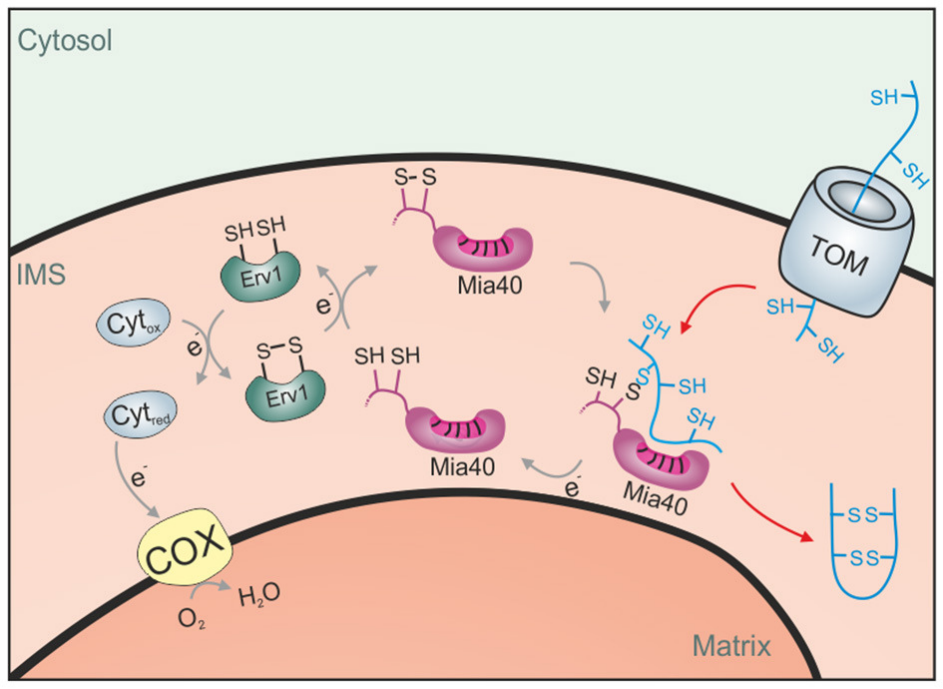
The disulfide relay system of the IMS. Proteins of the IMS enter the compartment through the TOM complex. They are typically of small size and contain several reduced cysteine residues. The IMS receptor/oxidoreductase Mia40 is able to form mixed disulfides with these proteins and promotes their oxidation. The FAD-containing sulfhydryl oxidase Erv1 maintains Mia40 in its oxidized form and can either transfer electrons directly to oxygen or use cytochrome *c* as an electron acceptor.

Mechanistically, the function of cytochrome *c* heme lyase for the import of cytochrome *c* is presumably similar to that of Mia40, a highly conserved IMS proteins found in mitochondria of plants, fungi, animals and humans. Mia40 is an oxidoreductase that can introduce disulfide bonds into its substrates and most IMS proteins indeed contain disulfide bonds (Gabriel et al., 2007; Longen et al., 2009; Kawamata and Manfredi, 2010; Klöppel et al., 2011; Vögtle et al., 2012; Kritsiligkou et al., 2017). Mia40 was initially identified in mitochondria of budding yeast (Sickmann et al., 2003; Chacinska et al., 2004; Naoe et al., 2004) before other orthologs were discovered. Mia40 is also referred to as Tim40 (in yeast) and as CHCHD4 (in mammalian cells). All Mia40 homologs share a highly conserved central region containing six invariant cysteine residues. A redox-sensitive CPC motif is essential for the oxidoreductase activity of Mia40. At steady state, this CPC is predominantly present in the oxidized state, although the degree of Mia40 oxidation might vary between different organisms and prevailing redox conditions (Bien et al., 2010; Sztolsztener et al., 2013; Kojer et al., 2014). The cysteine residues form intermolecular disulfide bonds with Mia40 substrates that can be stable for several minutes (Chacinska et al., 2004; Naoe et al., 2004; Mesecke et al., 2005; Longen et al., 2009; Sideris et al., 2009; Koch and Schmid, 2014c) and, at least *in vitro*, can promote both oxidation and isomerization reactions (Weckbecker et al., 2012; Koch and Schmid, 2014a). Erv1 (ALR in humans) is a FAD-bound sulfhydryl oxidase in the IMS that maintains Mia40 in its active, oxidized state (Lisowsky, 1994; Allen et al., 2005; Mesecke et al., 2005; Rissler et al., 2005; Terziyska et al., 2005; Ang and Lu, 2009; Tienson et al., 2009). Erv1 can either directly reduce oxygen to hydrogen peroxide or use cytochrome *c* as an electron acceptor (Figure 3). Alternatively, it can interact with the fumarate reductase Osm1 in order to get re-oxidized under anaerobic conditions (Neal et al., 2017).

## Mechanisms of Mia40-mediated protein import into the IMS

The CPC motif of Mia40 is in direct proximity to its hydrophobic substrate-binding pocket, which is formed by two anti-parallel helices stabilized by two structural disulfide bonds (Banci et al., 2009; Kawano et al., 2009). Mia40 substrates reach the IMS via translocation through the TOM pore and already bind to Mia40 during their translocation (von der Malsburg et al., 2011; Banci et al., 2012; Peleh et al., 2016). Mia40 recognizes specific patterns in its substrates referred to as intermembrane space targeting signal (ITS) or mitochondrial IMS–sorting signal (MISS) (Milenkovic et al., 2009; Sideris et al., 2009). However, the specificity of the Mia40 binding might be rather low since, at least *in vitro*, Mia40 interacts rather generally with hydrophobic protein stretches, particularly if they are of helical nature (Koch and Schmid, 2014c). In this reaction, Mia40 serves as a receptor that facilitates protein translocation by substrate trapping (Peleh et al., 2016). The interaction of Mia40 with its substrate can last from several seconds to minutes, and it was suggested that this prevents the back-translocation of Mia40 substrates into the cytosol.

**Figure 4:**
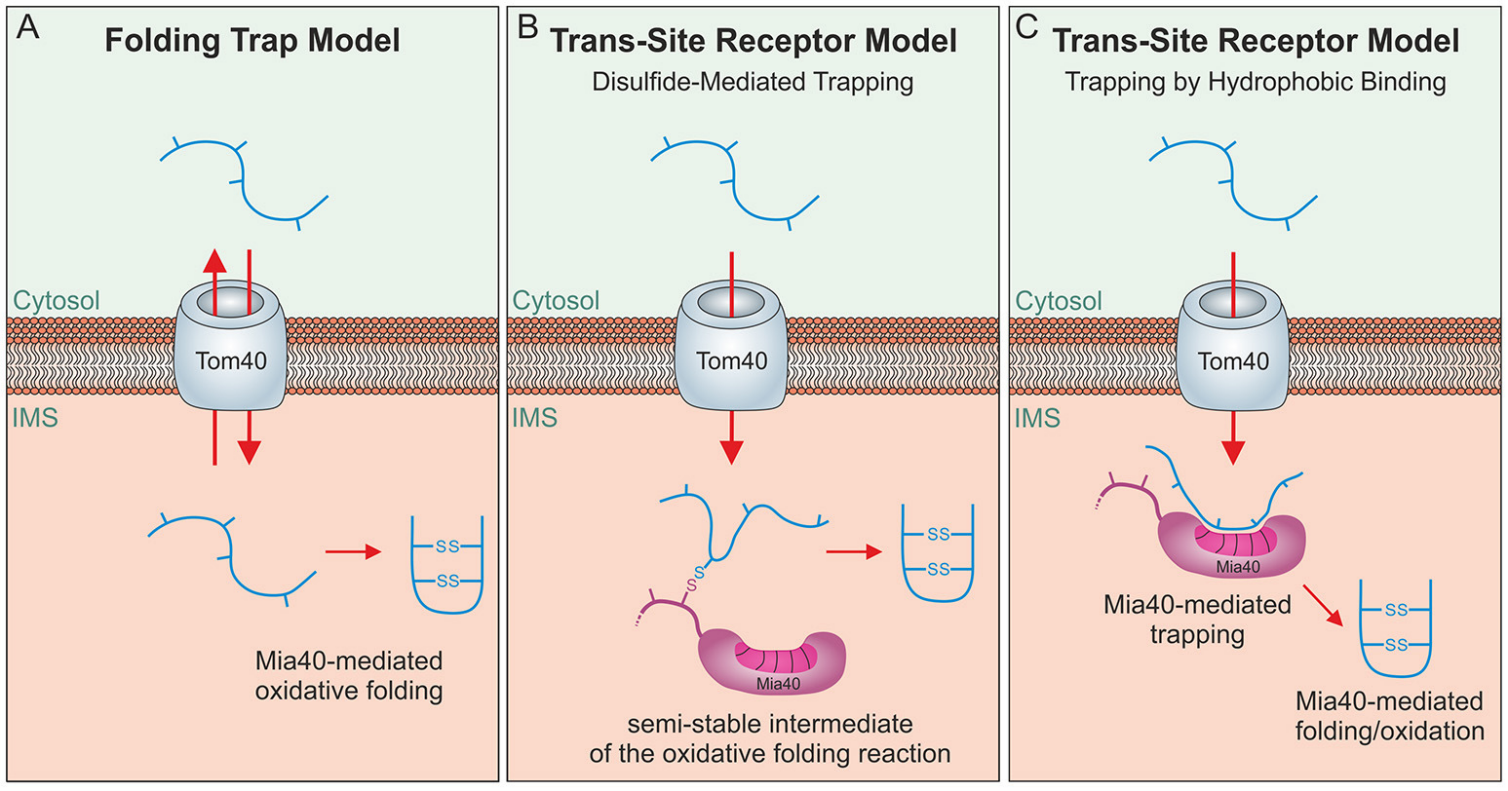
Different models of oxidative folding by Mia40. **(A)** Folding Trap Model: This model was inspired by the observation that reduced IMS proteins can back-translocate from the IMS to the cytosol. Since Mia40-mediated folding prevents this back-translocation, it was proposed that Mia40 does not directly promote translocation across the membrane but rather traps IMS proteins that were imported by facilitated diffusion through the TOM complex. **(B)** Disulfide-mediated trapping: Mia40 binds incoming proteins through mixed disulfides to prevent their backsliding into the cytosol and thus serves as a trans-site receptor that functions in a redox-mediated manner. **(C)** Trapping by hydrophobic binding: Mia40 is able to serve as a trans-site receptor that can mediate protein translocation in an oxidation-independent manner using hydrophobic interactions with the MIS/ITS signals in their sequence.

Mia40 substrates are released into the IMS in an oxidized state. The formation of disulfide bonds locks these proteins in a stably folded conformation thereby trapping them in the IMS as the folded proteins cannot pass the protein-conducting channel of the TOM complex (Sideris and Tokatlidis, 2007; Morgan and Lu, 2008; Bragoszewski et al., 2015). Initially, a “folding trap hypothesis” was proposed suggesting that newly synthesized IMS proteins would diffuse into and out of the IMS unless their oxidation by Mia40 keeps them within mitochondria (Allen et al., 2003; Lutz et al., 2003; Figure 4A). Indeed, even fully imported, endogenous IMS proteins were found to be released from the IMS through the TOM pore if their structural disulfide bonds are reduced by the addition of reductants (Bragoszewski et al., 2015). More detailed analyses of the import process and the use of site-specific cysteine mutants indicated that Mia40 serves as a molecular trap that binds incoming polypeptides via disulfide bonds (Figure 4B) to mediate their translocation through the TOM pore (Milenkovic et al., 2009; Sideris et al., 2009; von der Malsburg et al., 2011; Banci et al., 2012; Koch and Schmid, 2014c). However, this model was challenged by the observation that the redox-active CPC motif of Mia40 is dispensable for IMS import and only crucial subsequently for substrate folding (Baker et al., 2012; Weckbecker et al., 2012; Wrobel et al., 2013; Peleh et al., 2016; Ramesh et al., 2016). Thus, Mia40 can initially serve as trans-site receptor or holdase that promotes protein translocation across the TOM pore in an oxidation-independent process (Figure 4C).

However, Mia40 is certainly more than a simple receptor protein as it serves as a chaperone with foldase activity for a number of substrate proteins, a function which can even be observed for substrate proteins that do not contain cysteine residues (Weckbecker et al., 2012). The oxidative folding in the IMS is facilitated by glutathione (Bien et al., 2010; Kojer et al., 2012, 2014) and a number of redox enzymes, such as the peroxidase Gpx3 or thioredoxins, however, their specific contribution to the oxidative folding process is still not well understood (Vögtle et al., 2012; Kritsiligkou et al., 2017). In mammalian mitochondria, Mia40 forms a complex with the oxidoreductase apoptosis inducing factor (AIF) which tethers Mia40 to the inner membrane (Hangen et al., 2015).

The three different mechanisms shown in Figure 4 are not mutually exclusive. Mia40 obviously is able to trap incoming polypeptides both by its hydrophobic interaction to their MISS/ITS signal and by the formation of mixed disulfides. The observation that oxidative folding is critical to maintain some IMS proteins stable in the IMS certainly also argues for an oxidative folding trap function that is relevant for IMS proteins. The contribution of each of these mechanisms to the import process might also differ between substrates and physiological conditions.

## Energetics of the Mia40-mediated protein import into the IMS

Little is known about the mechanistic steps that drive the translocation of substrates of the mitochondrial disulfide relay across the outer membrane. There is no evidence for a co-translational import of IMS proteins in which the translation on the ribosome could promote the translocation through the TOM complex. As long as translation is not inhibited by antibiotics almost no cytosolic ribosomes are associated with the mitochondrial outer membrane (Gold et al., 2017).

**Figure 5:**
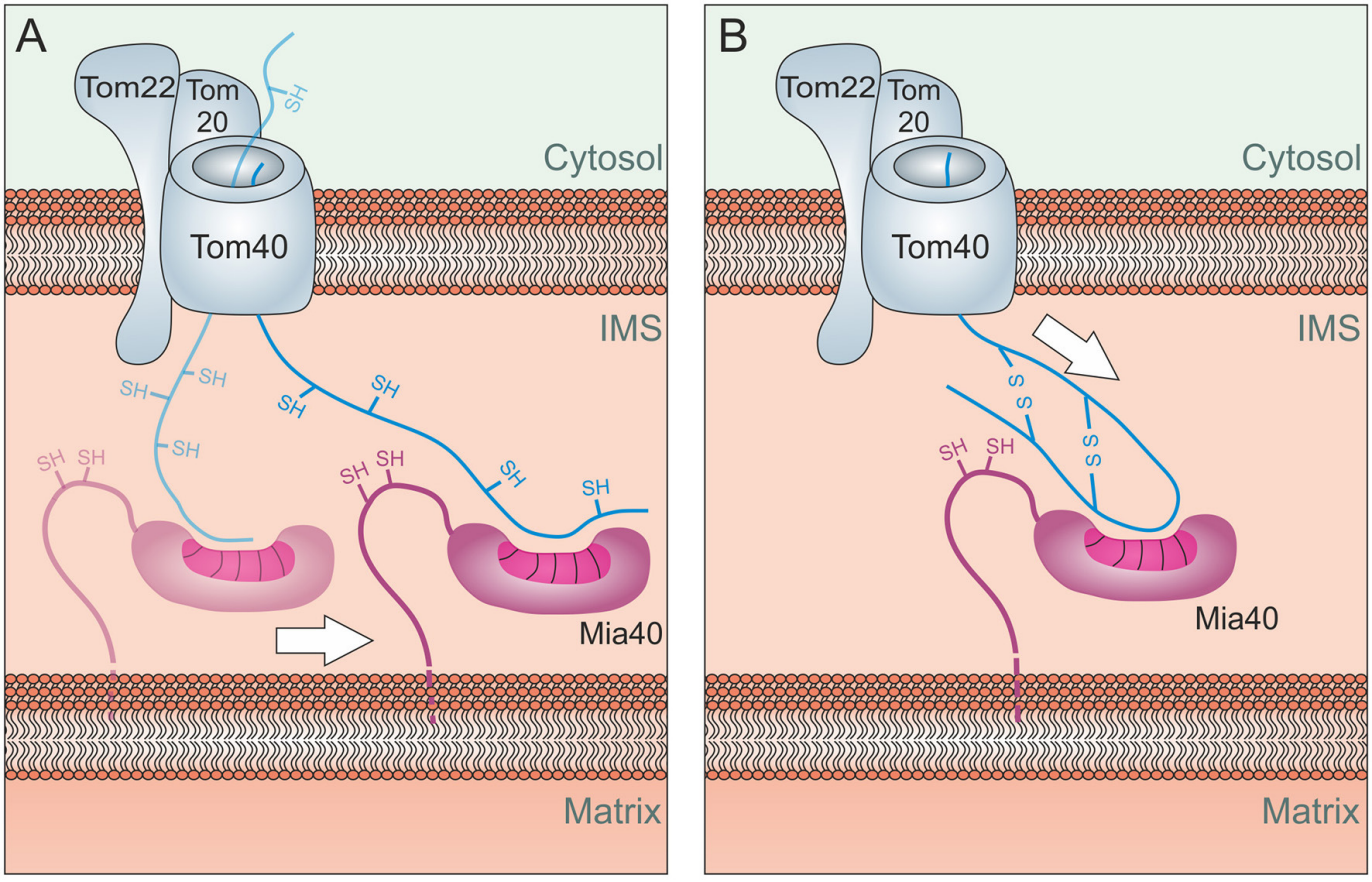
Energetics of Mia40-mediated import. **(A)** Lateral Diffusion: Mia40 binds to the translocating protein via hydrophobic binding which might be further stabilized by mixed disulfide bonds with the protein. Through lateral diffusion, Mia40 might drive the import reaction into the IMS. **(B)** Oxidation-mediated compaction: The oxidation of the incoming proteins and hence their compaction might contribute to the driving of the import process. Such a process would be limited to the import of short sequences across the TOM pore, explaining why most Mia40 substrates are of very small size.

Recent studies showed that the binding to Mia40 is essential for the translocation reaction but the oxidation of cysteines is not. This is supported by the observation that mutants of the Mia40 substrates Atp23, Tim9, and Tim10 accumulate in the IMS even if all cysteine residues are mutated (Baker et al., 2012; Weckbecker et al., 2012). However, these mutated proteins are rapidly degraded by the i-AAA protease Yme1 in the IMS. Moreover, a Mia40 mutant lacking the CPC motif still mediates protein import into the IMS, thus the oxidoreductase activity is not essential for its role as import component (Peleh et al., 2016). Mia40 might drive the import reaction by a lateral diffusion-mediated process (Figure 5A) similar to the process that drives import of inner membrane proteins (Figure 2C). Alternatively, Mia40 could simply act as a rectifier of diffusion by preventing backsliding of the substrate, comparable to the role of mtHsp70 in the Brownian Ratchet of the matrix (Figure 2A).

It is also feasible that the folding of substrate proteins by Mia40, for example due to the oxidation of substrates, promotes the compaction of IMS proteins and helps to translocate the termini of IMS proteins through the TOM pore (Figure 5B). However, such a process would presumably only be able to drive the translocation of relatively short segments into the IMS.

## The mechanism of Mia40-mediated protein import constrains the properties of IMS proteins

Even if the details are still not clear, it is obvious that in respect to its energetics, the Mia40-driven import differs considerably from the mtHsp70-driven translocation of matrix proteins which might explain several obvious differences in the molecular nature of matrix and IMS proteins:

### Mia40 substrates show a strong bias toward small size

The masses of most Mia40 substrates are extremely low (for example: Cmc1, 13.0 kDa; Cox17, 8.0 kDa; Cox19, 11.1 kDa; Mdm35, 9.7 kDa; Mic14, 13.8 kDa; Mrp10, 9.7 kDa; Tim8, 9.7 kDa; Tim9, 10.2 kDa; Tim10, 10.3 kDa; Tim12, 12.3 kDa; Tim13, 11.3 kDa). Many of these proteins have < 100 amino acid residues and therefore were initially not even annotated when the yeast genome was sequenced. The largest known soluble Mia40 substrate, Atp23 (32.2 kDa), differs from all other substrates since it has five rather than only two structural disulfide bonds and might employ several Mia40 molecules to be imported (Weckbecker et al., 2012; Kojer et al., 2014). In contrast, matrix proteins can be large and often of several distinct folding units (e.g., Pim1, 127 kDa; Kgd1, 114 kDa; Pet309, 113 kDa). The processive mtHsp70-mediated translocation obviously can easily mediate the import of polypeptides that consist of many hundreds of amino acid residues, but Mia40 might not. The small size of IMS proteins is not explained by the tiny lumen of this compartment since a number of IMS-exposed proteins that use presequences are much larger than Mia40 substrates (Yme1, 81.1 kDa; cytochrome b_2_, 65.5 kDa).

### Mia40 substrates are of simple helix-loop-helix structure

Almost all Mia40 substrates identified so far are consist of two short α-helices that are connected by two parallel disulfide bonds. Whereas substrates of the thioredoxin-based oxidation machineries of the ER and the periplasm are of very diverse structure, Mia40 substrates have a surprisingly consistent fold. This might be due to the poor catalytic capacity of Mia40 in oxidation and isomerisation reactions (Koch and Schmid, 2014a,b,c) but also might be used to drive the full translocation of proteins into the IMS during the oxidation process (Figure 5B). Obviously, such a process could only promote the translocation of a short stretch, thus explaining the small size of most IMS proteins.

### Mia40 substrates show only a low affinity for mitochondrial surface receptors

The receptors of the TOM complex efficiently bind to mitochondrial presequences but show only very low affinity to Mia40 substrates (Lutz et al., 2003). Instead of the cytosol-exposed TOM receptors, Tom70, Tom22, and Tom20, IMS proteins directly bind to the pore-forming subunit Tom40 and to the small TOM protein Tom5 (Kurz et al., 1999; Vögtle et al., 2012; Gornicka et al., 2014). It is unclear why IMS proteins avoid high-affinity interactions to the TOM complex, but it seems conceivable that the Mia40-dependent import process does not provide the energy required to release preproteins from the TOM receptors since this pathway functions independent of potent energy sources such as ATP hydrolysis or the membrane potential.

Apparently, the mitochondrial IMS is a very unique compartment in the cell as the limitations of the Mia40-mediated import systems constrained the properties of its proteome. The biochemical functions of many of the small helix-loop-helix proteins of the IMS are not understood and it will be very interesting to understand whether their common overall structure restricts their function to one common overall biochemical activity. However, one common type of reaction carried out by all of these different proteins is difficult to reconcile, given the many roles that these proteins exhibit in lipid homeostasis, respiratory chain complex assembly, or the transfer of copper ions.

## Final remarks

As far as we know the matrix-targeting machinery is very similar in different eukaryotes. There are certainly differences in the TOM receptors, which are not well conserved, however, the major components of the TOM and TIM23 complexes, as well as the import motor are conserved. In contrast, the mitochondrial disulfide relay differs considerably among eukaryotes and many protists lack a Mia40 homolog (though they contain an Erv1 protein). Also in plants, Erv1 can directly bind substrates making Mia40 dispensable. Unfortunately, little is known about the mechanisms of protein translocation in these organisms and other, so far not characterized factors might take over the holdase and foldase function of Mia40 (Carrie et al., 2010; Eckers et al., 2013; Haindrich et al., 2017; Peleh et al., 2017).

The mitochondrial intermembrane space developed from the bacterial periplasm during evolution. This common origin might explain the presence of disulfide bonds in most IMS proteins. The periplasm does not contain ATP (to avoid its loss by diffusion through porins of the outer membrane) which might have forced bacteria to develop a mechanism to fold periplasmic proteins by chaperones that act independently of ATP hydrolysis. DsbA, the thioredoxin that introduces disulfide bonds into periplasmic proteins, is one of these folding factors. The IMS is one of the very few eukaryotic compartments which, as far as we know, does not contain an Hsp70 chaperone system, perhaps because the early eukaryotic cells managed to exploit its oxidation machinery to drive protein translocation. Since the mitochondrial disulfide relay is of much lower complexity than the TIM23 import motor it might be possible to design a reconstituted system that can drive protein translocation. This certainly would be a big step forward in order to better understand its mechanistic properties in mitochondrial protein biogenesis.

## Author contributions

All authors listed have made a substantial, direct and intellectual contribution to the work, and approved it for publication.

### Conflict of interest statement

The authors declare that the research was conducted in the absence of any commercial or financial relationships that could be construed as a potential conflict of interest.
